# Factors associated with oral glucocorticoid use in patients with rheumatoid arthritis: a drug use study from a prospective national biologics registry

**DOI:** 10.1186/s13075-017-1461-3

**Published:** 2017-11-15

**Authors:** Rachel J. Black, Susan Lester, Rachelle Buchbinder, Claire Barrett, Marissa Lassere, Lyn March, Samuel Whittle, Catherine L. Hill

**Affiliations:** 1 Rheumatology Unit, Royal Adelaide Hospital, North Terrace, Adelaide, 5000 Australia; 2 Division of Medicine, The University of Adelaide, Adelaide, Australia; 3 Rheumatology Unit, The Queen Elizabeth Hospital, Woodville, Australia; 4 Monash Department of Clinical Epidemiology, Cabrini Institute, Melbourne, Australia; 5 Department of Epidemiology & Preventive Medicine, School of Public Health and Preventative Medicine, Monash University, Melbourne, Australia; 6 Redcliffe Hospital, Redcliffe, Australia; 7 School of Public Health and Community Medicine, University of New South Wales, Sydney, Australia; 8 Northern Clinical School, Institute of Bone and Joint Research, University of Sydney, Department of Rheumatology, Royal North Shore Hospital, Sydney, Australia

**Keywords:** Glucocorticoids, Rheumatoid arthritis, Epidemiology, Drug use

## Abstract

**Background:**

Glucocorticoids (GCs) are used in ~ 60% of patients with rheumatoid arthritis (RA). Although disease-modifying, they also have significant adverse effects. Understanding factors associated with GC use may help minimise exposure. The aims of the present study were to describe oral GC use in RA; determine any change in use over time; and determine factors associated with oral GC use, commencement or cessation.

**Methods:**

Adult patients with RA were identified in the Australian Rheumatology Association Database (ARAD), a national Australian registry that collects long-term outcome data from patients with inflammatory arthritis. Patients were categorised by their ARAD date of entry (DOE), with population-averaged logistic regression and transition state analysis used to determine any change in GC use over time. Fixed-effects panel regression was used to examine whether GC current use was associated with pain/arthritis activity/Health Assessment Questionnaire (HAQ) scores or medication use. Transition state analysis was used to assess whether these factors influenced the commencement or cessation of GCs.

**Results:**

A total of 3699 patients with RA completed a baseline ARAD questionnaire (73% female, mean age 57 years). The probability of GC use decreased over time according to ARAD DOE: September 2001 to March 2005, 55% (95% CI 52–58%); March 2005 to September 2008, 47% (45–49%); September 2008 to March 2012, 42% (39–45%); and March 2012 to October 2015, 39% (34–43%) (*p* < 0.001). Conventional synthetic disease-modifying anti-rheumatic drugs (OR 10.13; 95% CI 8.22–12.47), non-steroidal anti-inflammatory drugs (1.18; 1.02–1.37) and opioids (2.14; 1.84–2.48) were associated with GC current use, as were lower pain scores (0.94; 0.90–0.98), higher arthritis activity scores (1.09; 1.05–1.14) and poorer HAQ scores (1.52; 1.30–1.79). Use of biologic disease-modifying anti-rheumatic drugs (bDMARDs) was not associated with GC current use (0.98; 0.83–1.15) or GC cessation (HR 0.87; 95% CI 0.75–1.01), but it was associated with GC commencement (0.54; 0.47–0.62).

**Conclusions:**

The probability of oral GC use decreased over time, with reduced commencement and increased cessation of GCs. The modest effect of bDMARDs on GC cessation was not statistically significant.

**Electronic supplementary material:**

The online version of this article (doi:10.1186/s13075-017-1461-3) contains supplementary material, which is available to authorized users.

## Background

Glucocorticoids (GCs) are used in ~ 60% of patients with rheumatoid arthritis (RA) globally [1]. Although they have been shown to have disease-modifying properties [2], they are also associated with significant adverse effects [3, 4]. For this reason, many international guidelines recommend that the lowest possible dose and duration of GC therapy be used, if prescribed [5, 6]. Understanding the factors that are associated with GC use may help to minimise GC use. The use of GCs in RA may have changed over time with the introduction of biologic disease-modifying anti-rheumatic drugs (bDMARDs). Patients with severe disease resistant to conventional synthetic disease-modifying anti-rheumatic drugs (csDMARDs) prior to the introduction of bDMARDs may have been more likely to receive GCs than those with resistant disease and early access to bDMARDs. Previous studies have shown that bDMARDs can have a GC-sparing effect in RA, with a significant GC dose reduction seen in those commenced on bDMARDs compared with those who are not [7–11]. However, no prior studies have looked at the association between bDMARD use and GC cessation. Arguably, GC cessation rather than reduction should be the goal of therapy.

The following were the aims of the present study:

1. To describe the use of GCs among patients with RA enrolled in the Australian Rheumatology Association Database (ARAD) and any change in use over time
2. To determine factors associated with GC current use, including demographics, patient-reported pain score, arthritis activity score, Health Assessment Questionnaire (HAQ) score and concurrent medication use
3. To determine factors associated with the commencement and cessation of GCs in order to assess whether bDMARDs have a GC-sparing effect in this cohort

## Methods

### Population (ARAD)

ARAD is a voluntary Australian biologic registry established in 2001 to collect patient-reported long-term safety and other outcome data from patients with inflammatory arthritis, including RA, psoriatic arthritis, ankylosing spondylitis and juvenile idiopathic arthritis [12]. It includes participants commenced on bDMARDs as well as control subjects on conventional treatments (enrolment of control subjects since February 2007). Rheumatologists refer patients to the registry with minimal baseline information, including diagnosis, as well as rheumatoid factor and anti-citrullinated protein antibody (ACPA) status (ACPA collected since November 2010). All participants may commence, switch or cease bDMARDs at any point during their follow-up. Following written informed consent, participants complete a baseline ARAD questionnaire, with a follow-up questionnaire completed every 6 months for 2 years and then at 12-monthly intervals. The initial questionnaire is defined as the ‘baseline’ questionnaire, and there are no exclusions for disease duration, prior therapies or associated co-morbidities. The questionnaire was initially available in paper form only, but an electronic version has been available since August 2009. Adult participants with a diagnosis of RA were selected for this analysis, with data censored at October 2015. In order to reflect real-life clinical practice, participants are included in the registry with a diagnosis of RA based on expert clinical opinion (rheumatologist) rather than classification criteria. In Australia, bDMARDs can be prescribed for RA only by rheumatologists, and prescribing is restricted on the basis of the following criteria: (1) The patient has severe, active RA; (2) the patient has failed a 6-month intensive csDMARD trial with a minimum of two agents for a minimum of 3 months each; (3) the patient can demonstrate failure to achieve an adequate response to 6 months of intensive prior treatment by an elevated erythrocyte sedimentation rate > 25 mm/h and/or an elevated C-reactive protein level > 15 mg/L, and the patient has an active joint count of ≥ 20 active (swollen and tender) joints or ≥ 4 major active joints (elbow, wrist, knee, ankle, shoulder and/or hip). In Australia, there is universal access to medications via the Pharmaceutical Benefits Scheme (PBS). bDMARDs have been available on the PBS since August 2004. Prior to this, patients accessed bDMARDs through clinical trials.

Ethics approval for ARAD has been obtained from 18 committees and organisations across Australia (Additional file 1). This study was approved by The University of Adelaide Office of Research Ethics, Compliance and Integrity (approval number H-2015-258).

### Outcome measure

For oral GC current use, each questionnaire contains a section ‘medications for arthritis’ where patients indicate their use of oral GCs (prednisolone/prednisone) since their previous questionnaire as ‘never taken’, ‘currently taking’, ‘stopped taking’ or ‘don’t know’. Data regarding dosage are not collected. For this analysis, a time-varying ‘current use’ variable was created for which ‘currently taking’ was coded as ‘yes’ and ‘never taken’, ‘stopped taking’ and ‘don’t know’ responses were coded as ‘no’. The current use variable includes only oral GC use, with injectable GC use described but not included in the analyses.

### Predictors

Patient demographics, including age and sex, at baseline/initial questionnaire and a time-varying current age variable were considered as predictors in the analyses. Current use of bDMARDs and csDMARDs were coded as yes/no time-varying variables using the same method described for current oral GC use. Current use of bDMARDs included use of etanercept, adalimumab, anakinra, infliximab, rituximab, abatacept, tocilizumab, golimumab or certolizumab. Current csDMARD use included use of methotrexate, leflunomide, sulphasalazine, hydroxychloroquine, azathioprine, cyclosporin, intramuscular gold or penicillamine. Current use of non-steroidal anti-inflammatory drugs (NSAIDs) included use of celecoxib, diclofenac, ibuprofen, indomethacin, ketoprofen, meloxicam, naproxen, piroxicam or any other NSAID. Current use of opioids included use of aspirin and codeine, paracetamol and codeine, dextropropoxyphene, oxycodone, OxyContin, morphine or tramadol.

The ARAD questionnaire also contains a global evaluation of disease activity section in which patients are asked to indicate their level of pain and overall arthritis activity in the past week on a 0–100 visual analogue scale (0 indicates no pain/arthritis activity, and 100 indicates pain as bad as it could be/extreme arthritis activity). In addition to other measures of health-related quality of life, the questionnaire contains the HAQ [13]. HAQ scores range from 0 to 3, with higher scores reflecting greater disability [14]. These variables were also time-varying.

### Statistical analysis

Descriptive statistics were used to determine the patterns of oral GC use at baseline and throughout follow-up. It was hypothesised that GC use might vary according to the date of the baseline questionnaire. Prior to the availability of bDMARDs, there were limited treatment options for patients with RA with ongoing disease activity despite maximal csDMARD therapy. GC use may have been different in these patients who would have joined ARAD in the years closest to its inception, compared with those who joined in more recent years, when bDMARDs were more readily available. Population-averaged logistic regression (generalised estimating equation model) and transition state analysis were used to assess change in GC use over time, according to the date of baseline questionnaire. Date of entry (DOE) categories were created according to the date of the baseline questionnaire: 12 September 2001–15 March 2005, 15 March 2005–15 September 2008, 15 September 2008–15 March 2012, or 15 March 2012–6 October 2015.

A multivariable fixed-effects panel regression model was used to examine whether oral GC current use was associated with current age; disease duration; self-reported pain score; self-reported arthritis activity score; HAQ score; and current medication use, including bDMARDs, csDMARDs, NSAIDs and opioids. Age, self-reported pain score and self-reported arthritis activity score were transformed (divided by 10) for ease of interpreting the results. A fixed-effects model was chosen over a random effects model on the basis of the Hausman test. The fixed-effects model allows within-patient comparisons so that each patient is effectively acting as his or her own control.

Univariate transition state analysis was used to assess how these same factors influenced the HR of either commencing or ceasing oral GCs, with HRs relative to the first time point. In this analysis, two transition states were of interest: the transition from GC non-use at one visit to GC use at the next visit, and the transition from GC use at one visit to GC non-use at the next visit.

The fixed-effects panel regression model and transition state analyses included all patients with at least one follow-up visit after baseline. The panel regression model excluded those who were either on oral GCs at all visits or off oral GCs at all visits. Regression models were carried out using Stata version 12.1 software (StataCorp, College Station, TX, USA). The transition state analysis was carried out using R version 3.2.3 software (library msm version 1.6.4) [15, 16].

## Results

A total of 3699 ARAD participants with a diagnosis of RA completed a baseline questionnaire upon entry to ARAD, 73% of whom were female, with a mean age of 57 years (SD 13). Baseline characteristics of the cohort are shown in Table 1. At baseline 44% were taking an oral GC, 54% were taking a bDMARD, 74% were taking a traditional csDMARD, 43% were taking an NSAID and 32% were taking an opioid. There were 41% on combined bDMARD and csDMARD therapy, 13% on a bDMARD without csDMARDs, and 33% on csDMARDs without a bDMARD. Throughout follow-up (median 4 years, IQR 1.5–7 years), the prevalence of oral GC ever-use was 61%.

**Table 1:** Baseline characteristics of adult patients with rheumatoid arthritis enrolled in Australian Rheumatology Association Database

Baseline characteristics (*n* = 3699)	No. (%)^a^
Age, years, mean (SD)	57.1 (13.0)
Female sex	2761 (73.4%)
RF-positive^b^	2554/3083 (82.8%)
ACPA-positive^b^	162/239 (67.8%)
Disease duration, years, median (IQR)	10 (1–34)
Duration of ARAD follow-up, years, median (IQR)	4 (1.5–7)
Oral GC use	1641 (44.4%)
GC injection use	740 (20.0%)
bDMARD use	1983 (53.6%)
csDMARD use	2727 (73.7%)
bDMARD and csDMARD combined use	1517 (41.0%)
bDMARD use only (without csDMARD)	466 (12.6%)
csDMARD use only (without bDMARD)	1210 (32.7%)
Neither bDMARD nor csDMARD use	506 (12.7%)
NSAID use	1576 (42.6%)
Opioid use	1174 (31.7%)

*Abbreviations: ARAD* Australian Rheumatology Association Database, *RA* Rheumatoid arthritis, *RF* Rheumatoid factor, *ACPA* Anti-citrullinated protein antibody, *GC* Glucocorticoid, *bDMARD* Biologic disease-modifying anti-rheumatic drug, *csDMARD* Conventional synthetic disease-modifying anti-rheumatic drug, *NSAID* Non-steroidal anti-inflammatory drug

^a^Unless otherwise stated

^b^In those with known RF/ACPA status

### Change in GC use over time, according to ARAD date of entry

**Fig. 1:**
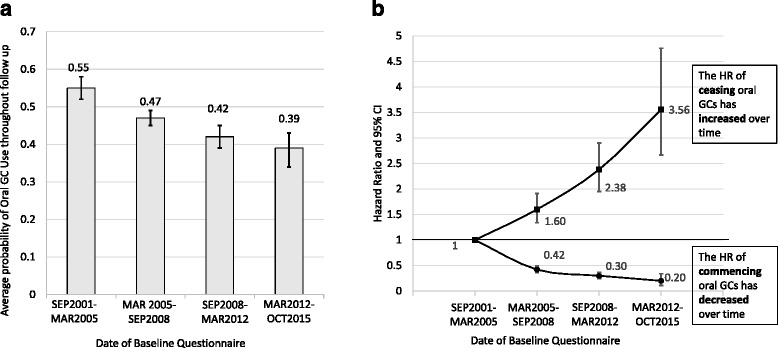
The association between glucocorticoid (GC) use and date of Australian Rheumatology Association Database baseline questionnaire. **a** Average probability of oral GC use throughout follow-up, according to date of baseline questionnaire. **b** The HR of commencing and ceasing an oral GC according to date of baseline questionnaire (HRs are relative to the first time point)

To test the hypothesis that GC use may vary over time, the probability of GC use throughout follow-up was examined according to DOE categories. The probability of oral GC use throughout follow-up deceased over time: September 2001 to March 2005, 55%; March 2005 to September 2008, 47%; September 2008 to March 2012, 42%; and March 2012 to October 2015, 39%, (*p* < 0.001) (Fig. 1a). In addition, the transition state analysis showed that the HR of commencing oral GCs compared with the first DOE category decreased with date of baseline questionnaire (March 2005 to September 2008 HR 0.42; September 2008 to March 2012 HR 0.30, March 2012 to October 2015 HR 0.20), and the HR of ceasing oral GCs increased (March 2005 to September 2008 HR 1.60, September 2008 to March 2012 HR 2.38, March 2012 to October 2015 HR 3.56) (Fig. 1b). Data from the transition state analysis can also be expressed as ‘sojourn times’, which is the average amount of time (in months) patients have spent in each state (Table 2).

**Table 2:** Sojourn times: mean amount of time (in months) spent on and off glucocorticoids, by Australian Rheumatology Association Database date of entry category

ARAD DOE category	State 1 (off GCs)		State 2 (on GCs)	
	Mean	95% CI	Mean	95% CI
12 Sep 2001 to 15 Mar 2005	57	52–63	151	130–175
15 Mar 2005 to 15 Sep 2008	137	123–152	94	86–104
15 Sep 2008 to 15 Mar 2012	193	159–234	63	56–72
15 Mar 2012 to 6 Oct 2015	292	172–493	42	33–54

*GC* Glucocorticoid

### Patient factors associated with oral GC current use

In the fixed-effects panel regression model (Table 3), longitudinal within-patient comparisons revealed that increasing age was associated with decreased GC current use (OR 0.24; 95% CI 0.07–0.81), but there was no association with disease duration (OR 1.05; 95% CI 0.93–1.19). Current use of bDMARDs was not associated with GC current use (OR 0.98; 95% CI 0.83–1.15); however, use of csDMARDs (10.13; 8.22–12.47), opioids (2.14; 1.84–2.48) and NSAIDs (1.18; 1.02–1.37) were all associated with increased GC current use. Higher current pain score (OR 0.94; 0.90–0.98) was associated with decreased GC current use, and higher arthritis activity scores (1.09; 1.05–1.14) and poorer HAQ scores (1.52; 1.30–1.79) were associated with increased GC current use.

**Table 3:** Multivariable fixed-effects panel regression model to determine factors associated with oral glucocorticoid current use at any time point

Factors associated with GC current use	OR	95% CI
Age, decades	0.24	0.07–0.81^a^
Disease duration, years	1.05	0.93–1.19
Current bDMARD use	0.98	0.83–1.15
Current csDMARD use	10.13	8.22–12.47^a^
Current NSAID use	1.18	1.02–1.37^a^
Current opioid use	2.14	1.84–2.48^a^
Self-reported pain score (10)	0.94	0.90–0.98^a^
Self-reported arthritis activity score (10)	1.09	1.05–1.14^a^
HAQ score (3)	1.52	1.30–1.79^a^

*Abbreviations: GC* Glucocorticoid, *bDMARD* Biologic disease-modifying anti-rheumatic drug, *csDMARD* Conventional synthetic disease-modifying anti-rheumatic drug, *NSAID* Non-steroidal anti-inflammatory drug, *HAQ* Health Assessment Questionnaire

The analysis included patients with rheumatoid arthritis with at least one follow-up visit after baseline and excluded those who were on oral GCs at all visits or off oral GCs at all visits (*n* = 1161). The fixed-effects model uses all available time points and allows for within-patient comparisons where each patient acts as his or her own control

^a^Indicates *p* < 0.05

### Patient factors associated with oral GC commencement and cessation

**Fig. 2:**
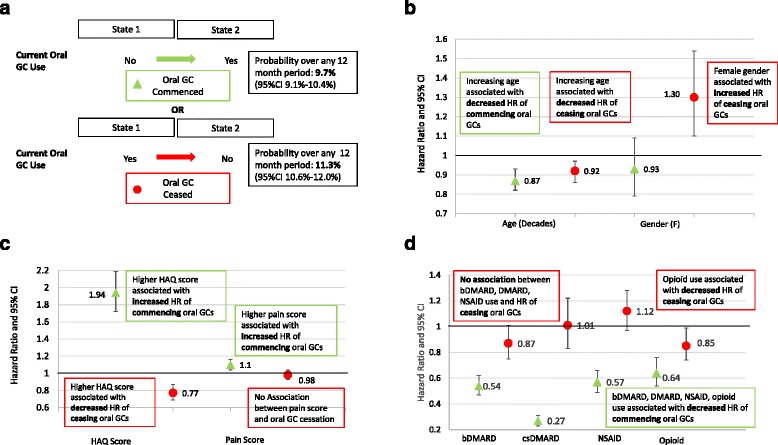
Transition state analysis of factors associated with the commencement and cessation of an oral glucocorticoid (GC). **a** Two transition states of interest are shown: (1) off oral GC at one time point, then on oral GC at the next time point (*green triangle* = oral GC is commenced), and (2) on oral GC at one time point, then off oral GC at the next time point (*red circle* = oral GC is ceased). The probability of commencing or ceasing an oral GC in any 12-month period is shown. **b** The association between age (decades) and sex (female) and the HR of commencing or ceasing an oral GC. **c** The association between Health Assessment Questionnaire (HAQ) score and pain score and the HR of commencing or ceasing an oral GC. **d** The association between concurrent medication use (biologic disease-modifying anti-rheumatic drug [bDMARD], conventional synthetic disease-modifying anti-rheumatic drug [csDMARD], non-steroidal anti-inflammatory drug [NSAID] and opioid) and the HR of commencing or ceasing an oral GC

In the transition state analysis (Fig. 2), within-patient comparisons revealed that increasing age was associated with decreased commencement and decreased cessation of oral GCs. Female sex was also associated with increased oral GC cessation. The moderate association between bDMARD use and oral GC cessation did not reach statistical significance. However, bDMARD, csDMARD or NSAID use was associated with a reduced HR of commencing oral GC therapy. Opioid use was associated with a reduced HR of both commencing and ceasing oral GCs. Higher HAQ score (greater disability) was associated with a greater HR of commencing oral GCs and a reduced HR of ceasing GCs. Higher pain scores were associated with an increased HR of commencing GCs, but there was no association between pain score and GC cessation. Higher arthritis activity score was not associated with either commencement or cessation of oral GCs.

## Discussion

In this study, we sought to describe the use of GCs amongst patients with RA over time and to determine factors associated with GC current use as well as GC commencement and cessation. In addition, we aimed to determine whether bDMARD use is associated with the cessation of GCs. This was carried out using data from patients with RA enrolled in ARAD, a longitudinal biologic registry.

In this RA cohort, the probability of GC use decreased over time and in recent years, the probability of commencing GCs had reduced, whereas the probability of ceasing GCs had increased. This potentially reflects an increasing awareness of GC-related adverse events (AEs) as well as increased availability of effective disease-modifying agents.

The influence of sex and increasing age on GC use was also of interest. In the panel regression model, increasing age was associated with a reduced HR of current GC use. In keeping with this, in the transition state analysis, we found that increasing age was associated with a reduced HR of commencing GCs, suggesting that clinicians are more cautious about commencing GC treatment in older patients. However, increasing age was also associated with a reduced HR of ceasing GCs, suggesting that, once started, it is more difficult to discontinue GC treatment in older patients. In the transition state analysis, females were more likely to cease GCs, which is in keeping with previous findings that females are more concerned than males about GC use [17].

In the panel regression model, patients had lower pain scores at times when they were on GCs than at times when they were not. Adding to this, the transition state analysis showed that individuals were more likely to commence GCs at times when their pain scores were higher, but that current pain scores had no influence on GC cessation. Given that GCs are effective anti-inflammatory agents in RA, it is not surprising that their use was associated with lower pain scores and that they were more likely to be commenced at times when pain scores were higher. The lack of association between lower pain scores and GC cessation may represent an opportunity for clinicians to reduce GC use; however, this would need to be assessed in the context of traditional disease activity scores.

The panel regression model also showed that patients had greater disability (as indicated by higher HAQ scores) at times when they were on GCs than at times when they were not. This raises the question whether GC use contributes to disability in RA, as has been shown in other rheumatic conditions such as systemic lupus erythematosus and ANCA-associated vasculitis [18–20]. The transition state analysis adds to our understanding of this, showing that patients with greater disability were more likely to commence GC therapy and less likely to cease therapy. Traditional measures of disease activity are not collected in ARAD; however, patients had slightly higher patient-reported arthritis activity scores when they were on GCs than when they were not. When considering the panel regression and transition state analysis together, it appears that the HAQ was a more important driver of oral GC use than pain or self-reported arthritis activity scores.

Patients were more likely to be taking GCs at times when they were also taking csDMARDs, NSAIDs or opioids than at times when they were not using these concurrent medications. However, concurrent bDMARD use was not associated with either increased or decreased current GC use. If it were assumed that current GC use reflects ongoing disease activity, then these findings would suggest that when patients were on csDMARDs, NSAIDs or opioids, their disease was more active than when they were not on these agents. Although this may seem to contradict our knowledge that csDMARDs reduce disease activity, in the setting of bDMARD use ongoing csDMARD use may indeed reflect patients with ongoing disease activity not controlled by bDMARD treatment alone. In the transition state analysis, the moderate association between bDMARD use and GC cessation did not reach statistical significance, suggesting that bDMARDs do not have a significant steroid-sparing effect in regards to GC cessation.

GC use is associated with many AEs, and the likelihood of these developing is influenced by total GC exposure (dose and duration of therapy) [21, 22]. GC cessation is therefore a clinically meaningful outcome when assessing the steroid-sparing effects of bDMARDs and other disease-modifying agents. Use of bDMARDs and csDMARDs was associated with a reduced HR of commencing GCs, which may be due to a reduced need for GCs because these agents are effective at controlling disease activity. NSAID or opioid use was also associated with a reduced HR of commencing GCs, and this may be because use of these agents reflects joint damage rather than active disease. Opioids were associated with a reduced HR of ceasing GCs. Patients on both opioids and GCs may represent a subgroup of patients with ongoing disease activity requiring GCs and joint damage leading to pain treated with opioids. It is plausible that it is more difficult to cease GCs in this subgroup of patients.

The main limitations of this study are that all data in ARAD are patient-reported, and neither GC dosage nor conventional measures of disease activity are collected. In addition, questionnaires are completed by patients at 6- to 12-monthly intervals and may therefore be associated with a recall bias. Enrolment in ARAD is done on an opt-in basis; therefore, there may be fundamental differences between those who do and do not choose to participate in the database. The ARAD questionnaire asks about GC use in the section ‘medications for arthritis’, and it is therefore assumed that the GC use reported has been prescribed for RA. However, many patients will have co-morbidities that are also indications for GCs, and it is possible that some of the reported GC use is driven by these co-morbidities. This could potentially bias the results towards the null hypothesis that there is no association between bDMARD use and GC cessation. The mortality in this sample was low, with 8% of the RA cohort recorded as deceased. Only limited data were available regarding cause of death; however, given the analyses made within-patient comparisons, it is unlikely that mortality would have significantly influenced the results.

Strengths of this study include the systematic and consistent way in which data are captured longitudinally in a real-life setting. In the treatment of RA in clinical practice, oral GCs may be given as short- or medium-term courses or used as a long-term therapy. Therefore, treatment may be started and stopped on numerous occasions throughout follow-up. Traditional methods for classifying GC use tend to be cross-sectional and do not capture the dynamic patterns of use that occur in clinical practice. For example, current use is often defined as use at a particular time point, such as at baseline or at the time of a predefined event (i.e., clinical remission or the development of an adverse effect). ARAD is a longitudinal dataset, allowing ‘current use’ to be defined as a time-varying indicator of whether a patient was taking oral GCs at each questionnaire time point. Most other relevant variables in the dataset were time-varying as well. The primary analyses (fixed-effects panel regression and transition state analysis) were specifically chosen in order to use the longitudinal nature of the data to determine within-patient concomitant predictors of both oral GC use and a change in use (commencement and cessation). This avoids the confounding due to unobserved/unmeasured variables that may occur in cross-sectional analyses.

## Conclusions

Oral GC use among Australian patients with RA participating in ARAD has decreased over time. Compared with patients who joined ARAD at its inception, those who joined the registry in more recent years had a lower probability of commencing GCs and a greater probability of ceasing GCs. Care needs to be taken when commencing oral GCs because it is often difficult to cease therapy once started, and bDMARD use has only a modest impact on this. Consideration of intramuscular and intra-articular GCs may help to offset oral GC use.

## Additional file

Additional file 1: ARAD list of current ethics approvals across Australia. (DOCX 19 kb)

## Abbreviations

ACPA: Anti-citrullinated protein antibody
AE: Adverse event
ARAD: Australian Rheumatology Association Database
bDMARD: Biologic disease-modifying anti-rheumatic drug
csDMARD: Conventional synthetic disease-modifying anti-rheumatic drug
DOE: Date of entry
GC: Glucocorticoid
HAQ: Health Assessment Questionnaire
NSAID: Non-steroidal anti-inflammatory drug
PBS: Pharmaceutical Benefits Scheme
RA: Rheumatoid arthritis
RF: Rheumatoid factor
