# Supplementary material for: Factors associated with oral glucocorticoid use in patients with rheumatoid arthritis: a drug use study from a prospective national biologics registry
Source: Arthritis Res Ther. 2017 Nov 15;19:253. doi: 10.1186/s13075-017-1461-3 (PMC5688724; doi:10.1186/s13075-017-1461-3)
Supplement: Additional file 1: — ARAD list of current ethics approvals across Australia. (DOCX 19 kb) [file 13075_2017_1461_MOESM1_ESM.docx]

**HUMAN RESEARCH ETHIC COMMITTEES THAT HAVE APPROVED ARAD**

**ACT Health & Community Care** **HREC** (EC 00100)

**AIHW - Australian Institute of Health & Welfare Ethics Committee** (EC 00103)

**Australian Government Department of Health HREC** (EC 00106)

**Cabrini Hospital HREC** (EC 00239)

**Cancer Council NSW** – **Cancer Institute Ethics Committee** (EC 00345)

**DOHWA – Dept. of Health WA HREC** (EC 00422)

**DVA - Dept. of Veterans' Affairs HREC** (EC 00366)
 **Monash University** **HREC** (EC 00234)

**North Sydney** **Local Health** **District HREC (Hawkesbury)** (EC 00132)

**NT HREC** **- NT Dept. of Health & Families & Menzies Sc** (EC 00153)

**Queensland HREC** (EC 00334)

**Royal Children's Hospital** **HREC** (EC 00238)

**SAC – Southern Adelaide Clinical HREC** - **SA Dept. of Health** (EC 00188)

**Southern Metropolitan Health Service HREC, Government of WA**

**St Vincent's Hospital** **(Melbourne)** **Ltd. HREC** (EC 00344)

**Tasmania** **Health & Medical HREC** (EC 00337)

**The Cancer Council Victoria** **HREC** (EC 00203)

**Women’s & Children’s Hospital SA Health Network HREC** (EC 00197)

**In Progress:**

**Royal Perth Hospital**

**No Longer active:**

**Princess Margaret Hospital, WA HREC** (EC 00268) – no longer active 2007-2016
